# Epigenome-wide DNA methylation profiling in septic and non-septic patients with similar infections: potential use as sepsis biomarkers

**DOI:** 10.3389/fcimb.2024.1532417

**Published:** 2025-01-24

**Authors:** Ian López-Cruz, José Luis García-Giménez, Manuel Madrazo, Judit García-Guallarte, Laura Piles, Federico V. Pallardó, Arturo Artero

**Affiliations:** ^1^ Department of Internal Medicine, Dr. Peset University Hospital, Valencia, Spain; ^2^ Department of Medicine, Faculty of Medicine & Dentistry, University of Valencia, Valencia, Spain; ^3^ Department of Physiology, Faculty of Medicine & Dentistry, University of Valencia, Valencia, Spain; ^4^ INCLIVA Biomedical Research Institute, Valencia, Spain; ^5^ Center for Biomedical Network Research on Rare Diseases (CIBERER), Institute of Health Carlos III, Valencia, Spain; ^6^ EpiDisease S.L., Parc Científic de la Universitat de València, Paterna, Spain

**Keywords:** sepsis, septic shock, DNA methylation, epigenetics, biomarkers, inflammation, immune system

## Abstract

**Introduction:**

Sepsis is a life-threatening condition caused by a dysregulated immune response to infection, leading to organ failure. Despite its significant global burden, the underlying mechanisms of immune dysfunction in sepsis remain incompletely understood. This study explores the role of DNA methylation in white blood cells in sepsis pathogenesis.

**Methods:**

A prospective case-control study was conducted to compare DNA methylation profiles between patients with community-acquired sepsis and matched controls who had similar infections but did not develop sepsis. Whole blood samples from these patients were analyzed using the Infinium MethylationEPIC v2.0 kit, enabling genome-wide methylation analysis. Selected genes with differential methylation were validated by pyrosequencing.

**Results:**

Significant differential DNA methylation patterns were identified between septic and non-septic individuals uising. Our results suggest that DNA methylation changes are closely linked to the pathophysiological processes of sepsis, influencing immune cell activation, inflammation, and organ dysfunction. The most prominent findings include the hypomethylation of immune-related genes (*SERPINA1, AZU1, MPO*, and *SLX4*), which were strongly correlated with clinical severity and inflammatory markers such as SOFA scores and PCT levels. Correlation analyses demonstrated significant associations between the methylation levels of these genes and clinical severity markers, such as SOFA score and PCT levels. Notably, *SLX4* hypomethylation showed the highest predictive value for poor prognosis (AUC 0.821), while *SERPINA1* hypomethylation exhibited strong diagnostic potential for sepsis (AUC 0.858).

**Discussion:**

Our results underscore the potential of DNA methylation changes, particularly in immune-related genes, to enhance the early detection of sepsis and to stratify patients based on severity. Future research should explore the therapeutic implications of these epigenetic alterations in sepsis care.

## Introduction

Sepsis is a clinical syndrome due to a dysregulated immune response to infection, which can lead to organ failure (Singer et al., 2016). Annually, 5 to 11 million people die from sepsis (Fleischmann et al., 2016; Rudd et al., 2020), making it a global health priority for the World Health Organization (WHO).

Pathogenesis of sepsis involves complex immune dysfunction including hyper-inflammatory response (“cytokine storm”) with systemic effects and immune suppression (Hotchkiss et al., 2013; Chen and Wei, 2021). This generalized inflammation can cause organ dysfunction through several mechanism such as apoptosis, pyroptosis, oxidative stress, mitochondrial dysfunction and microvascular thrombosis (Harrois et al., 2009; Gyawali et al., 2019; Danielski et al., 2020).

Epigenetic marks (including DNA methylation, post-translational histone modifications, and non-coding RNAs) play crucial roles in immune system regulation and response to infection (Delcuve et al., 2009; Beltrán-García et al., 2020). DNA methylation specifically influences cellular differentiation and the inflammatory response (Vachharajani and McCall, 2019; Beltrán-García et al., 2020). Studies in animal models link altered DNA methylation in gene promoter regions to hyper-inflammatory response and multi-organ failure during sepsis (Zhang et al., 2013; Singer et al., 2015; Huang et al., 2016; Shih et al., 2016; Cao et al., 2020). *In vitro* human monocyte studies have demonstrated that lipopolysaccharide (LPS) stimulation leads to hypomethylation of the tumor necrosis factor (TNF) gene promoter, which can be reversed by histone modifications (Gazzar et al., 2008; El Gazzar et al., 2010; Novakovic et al., 2016).

Despite these findings, clinical studies comparing epigenetic marks in sepsis are scarce. A study of 126 patients with community-acquired pneumonia found differences in DNA methylation enzyme expression, suggesting transcriptional dysregulation and chromatin reorganization (Hopp et al., 2018). Other studies comparing sepsis and non-infectious critical conditions reported distinct DNA methylation profiles affecting key genes for monocytes such as *IL1A*, *CCL22*, *CCR2*, and *STAT3* hypermethylation and *HLA-A*, *SOCS1*, *IL1R2*, and *CD46* hypomethylation (Lorente-Sorolla et al., 2019) as well as others genes related to immune response like *C3*, *ANG2*, *MPO* and *LPO* (Binnie et al., 2020). These findings suggest that DNA methylation changes could be useful as biomarkers in sepsis.

The heterogeneity of sepsis origins complicates its pathophysiological understanding, leaving the mechanisms by which the same infection triggers sepsis in some patients but not in others unknown. Beyond patient-specific factors like age and comorbidities, we propose investigating whether differential epigenetic responses may explain these variations. To address this, we conducted a prospective case-control study with two primary objectives: first, to identify DNA methylation differences between patients with community-acquired sepsis and those with the same infection source who did not develop sepsis; and second, to evaluate the potential of gene methylation levels as biomarkers for sepsis diagnosis or prognosis assessment.

## Materials and methods

### Study design

Prospective cohort study involving patients over 18 years old admitted to a University Hospital’s medical ward for community-acquired infections between September 2019 and December 2022. The goal was to analyze differential DNA methylation patterns between patients with sepsis and those with similar infections without sepsis.

Blood samples were collected in EDTA tubes within 24 hours of admission. DNA was isolated from leukocytes in whole blood samples and stored in the Biobank for Biomedical Research and Public Health of Valencia (IBSP-CV) for subsequent analysis.

Clinical and epidemiological data were obtained from medical history and electronic records, ensuring patient anonymity and confidentially. The study followed the principles of the Helsinki Declaration (Fortaleza version of 2013), the Spanish Laws on Personal Data Protection (15/1999) and Biomedical Research (14/2007). It was approved by the Ethics Committee of “Doctor Peset University Hospital” (Code: 73/19). All participants provided written informed consent.

### Patient selection

Patients admitted to the hospital in a medical ward with diagnosis of community acquired pneumonia (CAP), abdominal infections, or urinary tract infections (UTI) were consecutively evaluated. Patients admitted to a surgical ward were excluded, as well as patients with nosocomial infections. Other exclusion criteria included severe dependency (Barthel Index ≤35), pre-existing conditions with poor short-term vital prognosis, immunocompromised states or refusal to sign the informed consent.

Sepsis was diagnosed based on The Third International Consensus Definitions for Sepsis and Septic Shock (Sepsis-3) using qSOFA and SOFA scales (Seymour et al., 2016). Patients were categorized into a sepsis group (SOFA score ≥2) and a non-sepsis infection group (controls).

The control group consisted of patients who were also hospitalized for an acute community-acquired infection. They were matched with the cases based on the same infection source, the same sex, and an age difference of ±5 years. Standard clinical management was provided. Poor prognosis was considered if any of the following occurred: death during hospitalization or within the first 30 days after discharge, need for ICU admission, or readmission within 30 days of hospital discharge.

### DNA methylation analysis

Two approaches for DNA methylation analyses were conducted, consisting of the discovery phase using Infinium MethylationEPIC v2.0 Beadchip arrays (Illumina, San Diego, CA, USA). This approach enables high-throughput analysis to comprehensively identify differentially methylated positions (DMPs) and regions (DMRs) across the genome. Following this initial stage, a validation phase was conducted, focusing on the selection of candidate genes for analysis using bisulfite pyrosequencing. This targeted approach based on the analysis of the methylation profile of specific DMPs was selected because it offers a robust and cost-effective means of validating candidate biomarkers in a larger cohort. Importantly, this phase allows to strengthen the reliability of the findings by ensuring reproducibility of the results in a different sample set, while also correlating methylation changes with clinical parameters.

#### Genome-wide DNA methylation analysis by Infinium MethylationEPIC v2.0 BeadChip arrays

The discovery cohort consisted of 32 patients (16 sepsis, 16 controls), analyzed by whole genome amplification techniques and hybridization on Infinium MethylationEPIC v2.0 Beadchip arrays (Illumina, San Diego, CA, USA). These arrays contain probes for >935,000 CpG sites across genes and promoter regions, covering nearly all CpG sites in genes regulated by methylation, as described in projects such as ENCODE and FANTOM5.

Following the manufacturer recommendations, 500 ng of DNA normalized to a concentration of 10 ng/μL were treated with bisulfite conversion using an EZ DNA Methylation Kit (Zymo Research, Irvine, CA, USA). Afterwards, 4 µL of bisulfite-converted DNA was denatured, followed by isothermal amplification and fragmentation. Then, precipitation and resuspension phase optimally prepare the samples for hybridization process, which involved second denaturation in a thermal block at 95°C, transference to the arrays, and incubation in a hybridization chamber. Finally, the arrays were washed and assembled in flow chambers, where labeled nucleotides and antibodies were added to allow the distinction between methylated and non-methylated regions. The arrays were then read using an Illumina HiScan SQ (Illumina Inc, San Diego, CA, USA), generating images that were stored in.IDAT files for bioinformatic analysis.

#### Bioinformatic analysis

Data from Illumina arrays were processed using the minfi package in R, assessing quality [CpG percentage detection (0.05) ≥90%], normalizing data, and excluding probes with potential interference (Pidsley et al., 2016; Fortin et al., 2017). This included removing probes with low detection p-values, those overlapping SNPs, sex-linked probes, and cross-reactive probes. Differential methylation analysis identified differentially methylated positions (DMPs) and regions (DMRs) between septic and non-septic patients.

DMP analysis was performed using the limma package in R (Ritchie et al., 2015), with significance determined by adjusted p-value (false discovery rate, FDR) <0.05. DMRs were identified using the DMRcate package (Peters et al., 2015). Functional analysis linked DMPs and DMRs to Gene Ontology (GO) terms and Kyoto Encyclopedia of Genes and Genomes (KEGG) pathways, using the clusterProfiler package (Yu et al., 2012) to identify over-represented biological systems.

#### Validation of the DNA methylation analysis by bisulfite pyrosequencing

A second DNA methylation analysis was conducted by bisulfite pyrosequencing, focused on selected genes, aiming validation of previous results on an independent cohort with 28 patients (14 sepsis, 14 controls).

DNA was treated with bisulfite using the EpiTect Fast DNA Bisulfite Kit (Qiagen, Hilden, Germany). Then, PCR amplification followed by pyrosequencing assessed cytosine methylation levels in specific gene promoters. PyroMark PCR Kit (Qiagen) and customized primers were used. Standard pyrosequencing conditions and the PyroMark Q48 Autoprep software facilitated primer design and analysis. Primers used in pyrosequencing experiments are provided as **Supplementary Material** (**Supplementary Table S1**).

### Statistical analysis

Statistical analyses were conducted using IBM SPSS Statistics version 22. Normality was assessed with the Kolmogorov-Smirnov test. Means and standard deviations were calculated for normally distributions, while medians and interquartile ranges (IQR) were used for non-normal distributions. Qualitative variables were reported as counts and percentages. Comparisons were made using Student’s t-test or ANOVA for parametric data and Mann-Whitney U test for non-parametric data. Chi-square and Fisher’s exact tests were used for qualitative variables, and Spearman’s coefficient assessed correlations. A significance level of α=0.05 was applied.

## Results

### Patient demographics, clinical data and outcomes

During the study period, 789 patients were reviewed, however, only 60 met the inclusion and exclusion criteria and were ultimately included in the study. Flowchart is provided as **Supplementary Material** (**Supplementary Figure S1**).

Among the total patients in the sample, the median age was 78 years (IQR 68-84), with females accounting for 58.3% of the sample. Source of infection was UTI in 46 patients (76.7%), CAP in 12 patients (20%), and cholangitis in 2 patients (3.3%). Common comorbidities included diabetes mellitus (31.7%), chronic obstructive pulmonary disease (21.7%), chronic kidney disease (16.7%), and heart failure (15%). The Charlson Comorbidity Index had a median score of 5 points (IQR 4–6). Independence for activities of daily living (ADL) evaluated with the Barthel Index was seen in 42 patients (70%), mild dependence in 16 (26.7%), and moderate dependence in 2 (3.3%). FRAIL scale classified 23 patients (38.3%) as non-frail, 19 (31.7%) as pre-frail, and 18 (30%) as frail.

When the data from the 30 patients with sepsis and the 30 matched controls without sepsis were compared, no differences were found in most comorbidities, Charlson comorbidity index scores, polypharmacy criteria, dependency level according to the Barthel index, or frailty criteria. A history of previous neoplasia was more frequent in septic patients (20% vs. 1%, p=0.035). Septic patients showed a higher risk of malnutrition compared to controls (43.3% vs. 16.7%, p=0.024), based on their MNA scale scores. Aggregated data on comorbidities and functional status for patients from both cohorts about are shown in **Table 1**.

**Table 1:** Comorbidities and functional status of patients prior to hospital admission.

	Septic patients (n=30)	Non-septic patients (n=30)	p-value
Comorbidities, n (%)			
Stroke	1 (3.3)	1 (3.3)	1.000
Asthma	1 (3.3)	5 (16.7)	0.073
Dementia	1 (3.3)	1 (3.3)	0.236
Diabetes mellitus	9 (30)	10 (33.3)	0.781
Coronary artery disease	3 (10)	4 (13.3)	0.687
Chronic kidney disease	4 (13.3)	6 (20)	0.488
Connective tissue disease	2 (6.7)	1 (3.3)	0.550
COPD	4 (13.3)	9 (30)	0.113
Chronic liver disease	1 (3.3)	2 (6.7)	0.550
Heart failure	6 (20)	3 (10)	0.274
Neoplasm	6 (20)	1 (3.3)	0.035
Peptic ulcer	1 (3.3)	3 (10)	0.290
Peripheral vascular disease	2 (6.7)	1 (3.3)	0.550
Charlson Comorbidity Index, median [IQR]	5 [4-6.25]	4.5 [4-6.25]	0.782
Polypharmacy ≥5 drugs, n (%)	20 (66.7)	19 (63.3)	0.787
Barthel Index, n (%)			
Independence ADL: 100 points	21 (70)	21 (70)	1.000
Mild dependence ADL: 60-95 points	8 (26.7)	8 (26.7)	1.000
Moderate dependence ADL: 40-55 points	1 (3.3)	1 (3.3)	1.000
FRAIL Scale, n (%)			
Not frail: 0 points	9 (30)	14 (46.7)	0.184
Pre-frail: 1-2 points	12 (20)	7 (1.7)	0.165
Frail: 3-5 points	9 (30)	9 (30)	1.000
MNA Scale, n (%)			
Normal nutritional status: 12-14 points	17 (56.7)	24 (80)	0.052
Malnutrition risk: 8-11 points	13 (43.3)	5 (16.7)	0.024
Malnutrition: ≤ 7 points	0	1 (3.3)	0.236

ADL, activities of daily living; COPD, chronic obstructive pulmonary disease; FRAIL, fatigue, resistance, aerobic capacity, illnesses and loss of weight; MNA, Mini Nutritional Assessment.

Significant differences were observed in qSOFA and SOFA scores, septic shock and SIRS criteria, and APACHE-II scores. Septic patients exhibited higher heart and respiratory rates, and lower blood pressure and oxygen saturation levels compared to controls. Glasgow Coma Scale scores were also lower in septic patients. Septic patients had elevated serum creatinine, C reactive protein (CRP), procalcitonin (PCT) and lactate levels, while hemoglobin, platelets count, and CD4-lymphocytes count were lower compared to controls. Severity scales and clinical characteristics are shown in **Table 2**.

**Table 2:** Severity scales and clinical characteristics at hospital admission.

	Septic patients (n=30)	Non-septic patients (n=30)	p-value
Severity scales			
qSOFA ≥2, n (%)	26 (86,7)	0	**<0,001**
SIRS, n (%)	30 (100)	14 (46,7)	**<0,001**
Severe sepsis, n (%)	26 (86,7)	1 (3,3)	**<0,001**
Septic shock, n (%)	3 (10)	0	0,236
SOFA, median [IQR]	4 [3-5]	0 [0-1]	**<0,001**
APACHE-II, mean ± SD	13,6 (± 2,9)	7,4 (± 3,2)	**<0,001**
Glasgow score ≤14, n (%)	14 (46,7)	1 (3,3)	**<0,001**
Vital signs			
Temperature, mean ± SD (°C),	37,7 (± 1,2)	37,2 (± 1,1)	0,129
SBP, mean ± SD (mmHg)	106 (± 29)	136 (± 21)	**<0,001**
DBP, mean ± SD (mmHg)	59 (± 13)	77 (± 14)	**<0,001**
MAP, mean ± SD (mmHg)	75 (± 18)	97 (± 15)	**<0,001**
HR, mean ± SD (lpm)	108 (± 20)	89 (± 19)	**<0,001**
RR, median [IQR] (rpm)	24 [22-27]	18 [16-20]	**<0,001**
SatO2, median [IQR]	93 [91-96]	96 [94-98]	**0,010**
SatO2/FiO2, median [IQR]	438 [424-457]	457 [448-462]	**0,004**
Laboratory parameters			
Creatinine (mg/dl), median [IQR]	1,75 [1,30-2,92]	0,93 [0,75-1,27]	**<0,001**
Bilirubin (mg/dl), median [IQR]	0,7 [0,45-1,30]	0,7 [0,5-1]	0,853
Albumin (mg/dl), mean ± SD	3,4 (± 0,5)	3,8 (± 0,4)	**0,004**
Na^+^ (mEq/l), mean ± SD	137 (± 3)	137 (± 4)	0,701
K^+^ (mEq/l), mean ± SD	4,2 (± 0,8)	4,1 (± 0,5)	0,677
Hemoglobin (g/l), median [IQR]	12,2 [11,4-12,8]	13 [11,3-14,4]	**0,025**
Hematocrit (%), mean ± SD	36 (± 5)	39 (± 5)	**0,024**
PaO2/FiO2*, mean ± SD	310 (± 72)	326 (± 38)	0,659
pH, mean ± SD	7,39 (± 0,07)	7,40 (± 0,05)	0,402
Bicarbonate (mEq/L), mean ± SD	23 (± 4)	27 (± 2)	**<0,001**
Lactate (mmol/L), median [IQR]	1,9 [1,1-3,4]	1,35 [1,1-1,9]	**0,020**
CRP (mg/l), mean ± SD	285 (± 118)	163 (± 110)	**<0,001**
PCT (ng/ml), median [IQR]	19,8 [7,1-33,6]	0,6 [0,2-3,3]	**<0,001**
Leukocytes (cel/mm^3^), median [IQR]	14.950 [11.825-19.950]	12.450 [9.225-18.400]	0,196
Lymphocytes**, median [IQR]			
-CD4 lymphocytes	268 [167-415]	521 [260-828]	**0,017**
-CD8 lymphocytes	136 [84-229]	196 [142-440]	0,063
-CD4/CD8 ratio	1,61 [1-2,87]	1,80 [1,39-3,8]	0,521
-CD19 lymphocytes	91 [43-141]	83,5 [65-147]	0,441
-NK lymphocytes	94 [56-202]	177 [95-234]	0,059
Platelets (cel/mm^3^), median [IQR]	146.000 [120.000-203.500]	201.500 [171.000-249.000]	**0,009**

bpm, Beats per minute; CRP, C reactive protein; DBP, Diastolic blood pressure; HR, Heart rate; MAP, Mean arterial pressure; PaO_2_/FiO_2_, Oxygen arterial pressure/Fraction of inspired oxygen ratio; PCT, procalcitonin; RR, Respiratory rate; rpm, Breaths per minute; SBP, Systolic blood pressure. *n=19; **n=47.Bold values are indicated to highlight the statistically significant values.

The patients were grouped into two cohorts for the differential methylation analysis: the discovery cohort (32 patients) and the validation cohort (28 patients). A comparison of their basal characteristics was performed. No significant differences were found in age, sex, severity according to the SOFA score, Charlson comorbidity index, degree of dependence, or frailty criteria between both cohorts. No differences were observed in any of the analyzed comorbidities, including a history of neoplasia, or in the malnutrition criteria according to the MNA scale. The only difference observed was in serum procalcitonin levels within the first 24 hours, which were slightly higher in the discovery cohort (17,6 ng/ml vs. 12,1ng/ml; p-value 0,028). No differences were observed in other inflammatory markers or in any of the other laboratory parameters.

Pathogenic microorganisms were isolated in 46 patients (76.7%) of the total study cohort. In urinary tract infections, microbiological isolation was achieved through urine culture in 40 patients (87%) and through blood culture in 12 cases (26%). *Escherichia coli* was the most frequently isolated pathogen (68%), followed by *Klebsiella* spp. (19.4%) and other enterobacteria. There was only one case of urinary tract infection caused by *Pseudomonas aeruginosa* and one case caused by *Enterococcus faecalis*. Concordance between urine and blood culture results was observed in 11 patients, whereas one patient showed a positive blood culture and a negative urine culture. Seven cases of multidrug-resistant infections were identified among the urinary tract infections, four of which were extended-spectrum beta-lactamase (ESBL)-producing *E. coli* strains. In respiratory infections, *Streptococcus pneumoniae* was identified in 2 cases (16.7%) through antigen detection in a urine sample, and Influenza A virus was identified in 2 other cases (16.7%) via PCR on nasopharyngeal swabs, where bacterial superinfection was suspected. In abdominal infections, *Streptococcus anginosus* was isolated through blood culture in 1 case (50%).

Length of hospital stay was longer in septic patients compared to controls (5.5 [IQR 4–9.25] vs 3 days [IQR 2-5.25], p=0.001). Eight patients required ICU admission, with 7 (23.3%) in sepsis group and 1 (3.3%) in the non-sepsis group (p=0.052). The median ICU stay was 3 days [IQR 2–4.75]. Two patients died during the hospitalization, one in each group. The death of the non-septic patient was attributed to nosocomial pneumonia complicated by sepsis and organ failure, necessitating ICU admission.

### Generalized genome hypomethylation in septic patients

**Figure 1:**
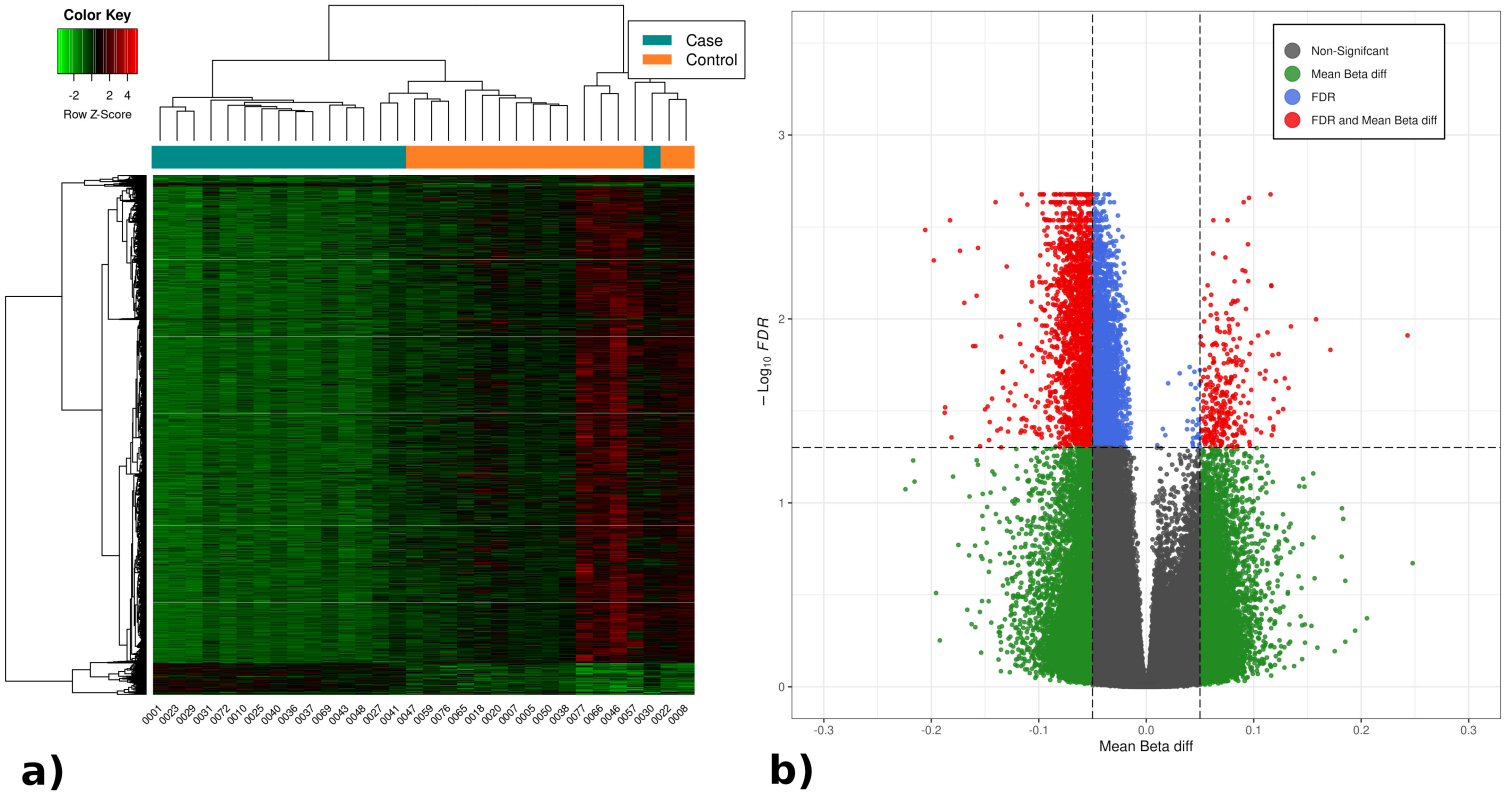
**(A)** Heatmap with the significant DMPs in the differential methylation analysis between septic and non-septic patients. **(B)** Volcano plot representing the results of the DMPs differential methylation analysis between septic and non-septic patients. The heatmap shows generalized hypomethylation (light green) in septic patients, while non-septic patients display varying degrees of hypo- and hypermethylation (green, black, red). Notably, patients cluster according to methylation levels, with septic patients mostly on the left and controls on the right (orange). In the volcano plot, all methylation positions are shown, with significant ones (FDR <0.05) above the horizontal line. DMPs with over 5% differential methylation appear outside the vertical lines, identifying 4,640 relevant DMPs: 4,323 hypomethylated (left) and 317 hypermethylated (right).

A discovery cohort of 32 patients was analyzed using Infinium Methylation Array EPIC v2.0 assays. A total of 4,640 significant differentially methylated positions (DMPs) were identified (FDR<5%), of which 4,323 were hypomethylated and 317 were hypermethylated. From these, the 330 DMPs with a mean β-value difference between groups >8.5% were selected. A heatmap of the identified DMPs and their differential methylation values is displayed in **Figure 1**, along with a volcano plot illustrating the differential methylation analysis and FDR for all DMPs.

Subsequently, 85 statistically significant differentially methylated regions (DMRs) with a mean methylation difference >5% were found (72 hypomethylated and 13 hypermethylated). All significant DMPs and DMRs in this analysis are provided in a table as **Supplementary Material** (**Supplementary Tables S2**, **S3**).

### DNA methylation patterns are involved in essential processes related to immune system

**Figure 2:**
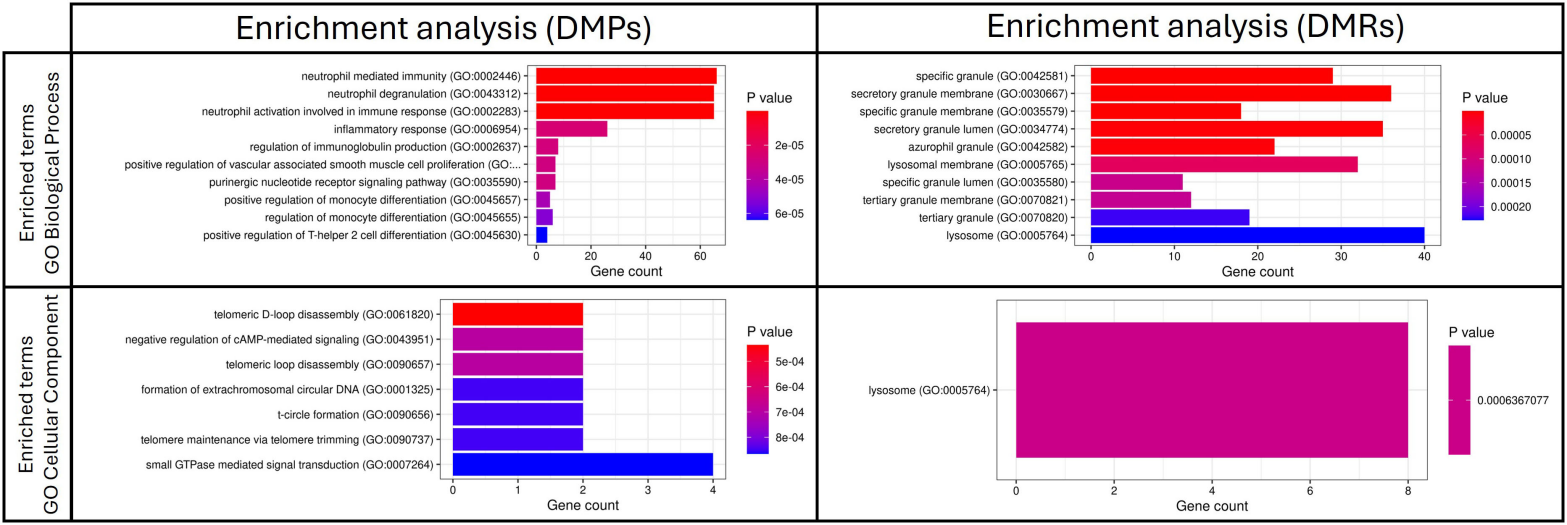
Top 10 significant GO terms obtained from the ORA analysis based on significant DMPs and DMRs in the comparison between patients with sepsis and patients without sepsis. DMPs, differentially methylated positions; DMRs, differentially methylated regions.

Over-representation analysis (ORA) of genes annotated to the significant DMPs and DMRs identified 25 significant Gene Ontology (GO) Biological Process terms and 23 significant GO Cellular Component terms from DMPs, while 7 significant GO Biological Process terms and 1 significant GO Cellular component term from DMRs. No GO Molecular Function term or Kyoto Encyclopedia for Genes and Genomes (KEGG) pathways were identified. **Figure 2** illustrates the top ten significant GO terms obtained from ORA. All enrichment analysis results are provided as **Supplementary Material** (**Supplementary Table S4**).

Key biological processes included neutrophil activation, degranulation, monocyte differentiation, immunoglobulin production and Th2 differentiation. Cellular components related to neutrophil-specific vesicles (azurophilic, secondary and gelatinase granules) were also identified.

Based on their high differential methylation levels and their role in the immune functions identified, four genes (*SERPINA1*, *AZU1*, *MPO* and *SLX4*) were selected for further validation, supported by available evidence of their biological roles in sepsis.

### Validation of SERPINA1, AZU1, MPO and SLX4 hypomethylation and correlation with clinical variables

Bisulfite pyrosequencing was used to validate methylation patterns in the remaining 28 patients, forming an independent validation cohort, as well as in most patients from the discovery cohort, except for two samples that could not be analyzed due to the exhaustion of DNA from prior analyses. Significant hypomethylation was observed in all four genes across both cohorts, confirming the initial findings (**Table 3**).

**Table 3:** Differential methylation analysis by bisulfite pyrosequencing for each cohort of the study.

Gen - Position	Discovery cohort (n = 30)*		Validation cohort (n = 28)		Total (n = 58)*	
	Met. mean diff. (%)	p-value	Met. mean diff. (%)	p-value	Met. mean diff. (%)	p-value
*SERPINA1* CpG1	-19,2	0,002	-11,74	0,025	-14,3	<0,0001
*AZU1* CpG1	-7,65	0,001	-2,96	0,049	-5,3	<0,0001
*AZU1* CpG2	-9,55	0,002	-3,34	0,042	-5,4	<0,0001
*MPO* CpG1	-6,44	0,007	-3,17	<0,001	-4,5	<0,0001
*MPO* CpG2	-6,75	0,011	-3,04	0,025	-4,5	<0,0001
*SLX4* CpG1	-9,83	0,003	-5,28	0,025	-5,6	0,0002
*SLX4* CpG2	-10,64	0,002	-4,25	0,034	-5,3	<0,0001
*SLX4* CpG3	-8,56	0,005	-3,14	0,033	-4,1	<0,0001

Met. mean diff., Methylation mean difference. Negative values stand for hypomethylation in the sepsis group.

*Two samples from the discovery cohort could not be analyzed by pyrosequencing due to the depletion of available DNA in the initial analysis.

**Figure 3:**
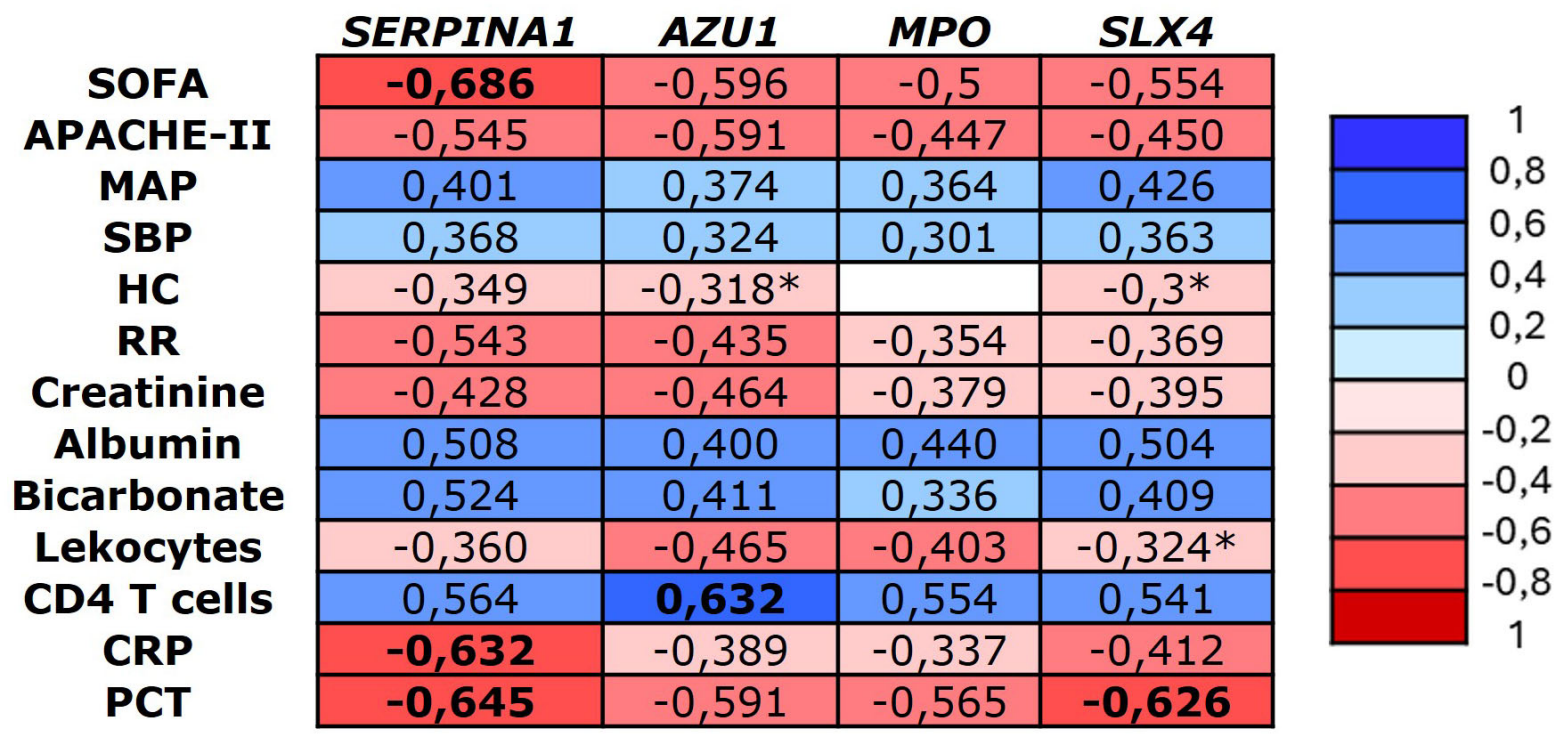
Heatmap representing the significant Spearman correlation coefficients (-1 to +1) between the identified genes and major clinical variables (p < 0.001). CRP, C reactive protein, MAP, Mean arterial pressure, HR, heart rate, PCT, procalcitonin, RR, respiratory rate, SBP, Systolic blood pressure. *Significant correlation p-value < 0.05.

Correlation analysis showed significant associations between methylation levels and clinical variables related to sepsis severity, inflammation, and organ failure. The strongest correlations were observed with the SOFA score at admission and PCT levels within the first 24 hours. Additionally, we found a strong positive correlation between the methylation levels of all the genes and CD4 T cell levels, particularly with *AZU1*. No significant correlations were found with temperature, Glasgow Coma Scale, hemoglobin, bilirubin, platelet count, pH, or lactate. All significant correlations (p-value <0.01) are shown in **Figure 3**.

### Hypomethylation of SERPINA1, AZU1, MPO and SLX4 are potential biomarkers in sepsis

**Figure 4:**
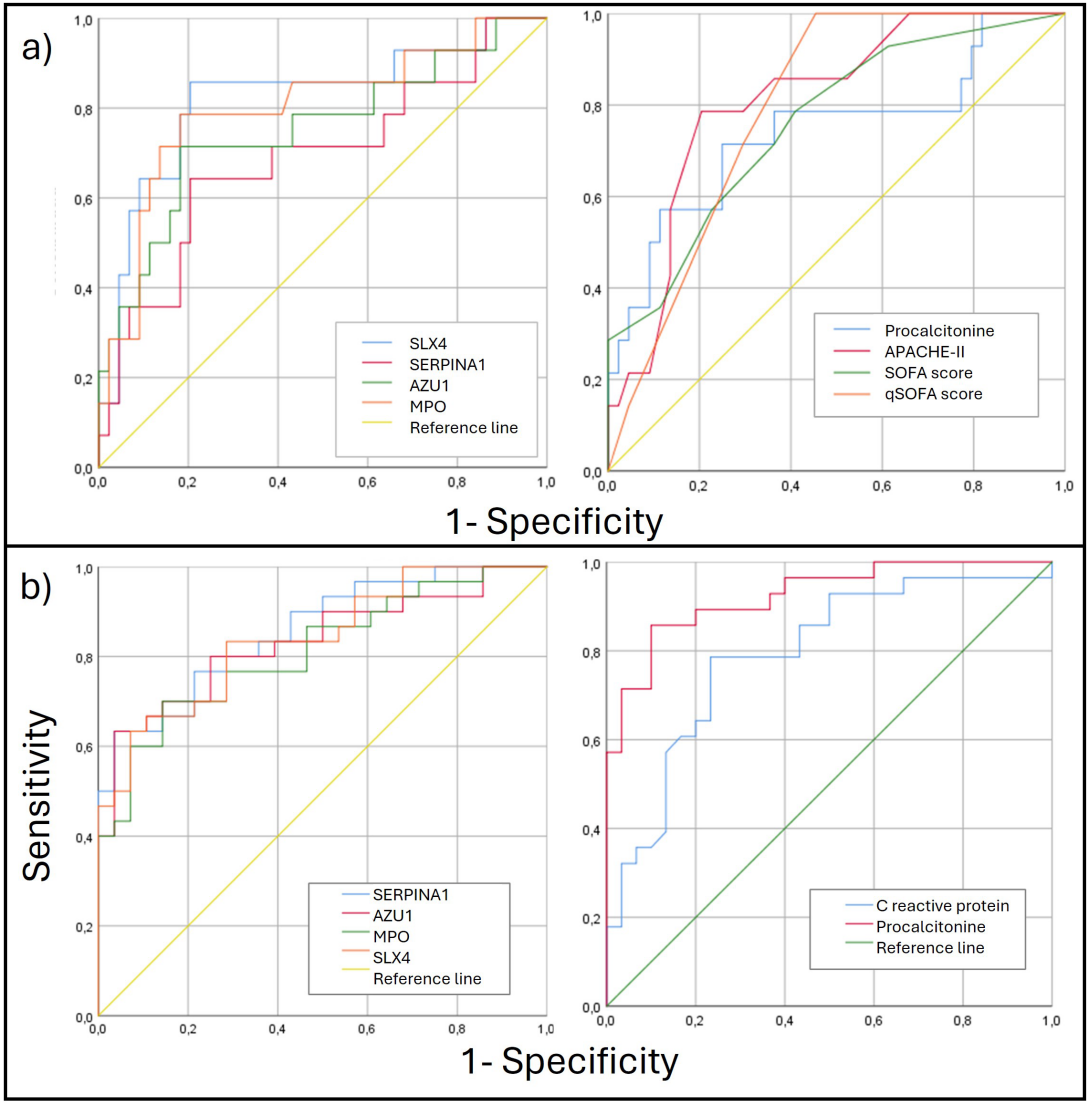
**(A)** ROC curves for poor prognosis discrimination for methylation genes levels (left) and clinical parameters (right). **(B)** ROC curves for diagnosis discrimination for methylation genes levels (left) and clinical parameters (right). ROC, Receiver Operating Characteristic.

Area under the curve (AUC) analysis of methylation levels for poor prognosis showed significant results for the validated four genes, with *SLX4* showing the best performance (AUC 0.821; CI: 0.678-0.964), followed by *MPO* (AUC 0.801; (0.655-0.947). For sepsis diagnosis, *SERPINA1* demonstrated the best performance (AUC 0.858; CI: 0.766-0.951) and all the 4 genes outperformed CRP. The AUC was also calculated for the clinical scoring systems (APACHE-II, SOFA, and qSOFA) and laboratory biomarkers (PCT, CRP). The prognostic and diagnostic AUC results are presented in **Table 4** and **Figure 4**.

**Table 4:** AUC and optimal cutoff points with their corresponding validity indexes for methylation genes levels, severity scores and biomarkers.

	Poor prognosis discrimination				Sepsis diagnosis discrimination			
	AUC (95% CI)	Cutoff	Se (%)	Sp (%)	AUC (95% CI)	Cutoff	Se (%)	Sp (%)
*SERPINA1*	0,688 (0,515-0,861)	≤12.39	64	80	0,858 (0,766-0,951)	≤17.01	77	79
*AZU1*	0,752 (0,589-0,914)	≤5.03	71	82	0,832 (0,726-0,938)	≤7.14	80	75
*MPO*	0,801 (0,655-0,947)	≤5.31	79	82	0,813 (0,704-0,923)	≤7.37	77	71
*SLX4*	0,821 (0,678-0,964)	≤7.18	86	80	0,839 (0,739-0,939)	≤9.38	83	71
SOFA	0,756 (0,611-0,902)	≥3	71	67	–	–	–	–
qSOFA	0,786 (0,672-0,900)	≥2	71	70	–	–	–	–
APACHE-II	0,796 (0,671-0,921)	≥12	79	77	–	–	–	–
PCT	0,742 (0,574-0,910)	≥6.15	79	63	0,925 (0,859-0,991)	≥3.62	89	80
CRP	–	–	–	–	0,793 (0,739-0,939)	≥226	79	77

AUC, Area under the curve; CRP, C reactive protein; PCR, procalcitonin; Se, sensitivity; Sp, specificity.

## Discussion

This study analyzed DNA methylation changes during sepsis and evaluated its potential as a biomarker. We included patients hospitalized with community-acquired infections, ensuring similar baseline characteristics to compare those who developed sepsis with those who did not. The exclusion criteria were designed to avoid confounding factors related to comorbidities or baseline conditions that might complicate the interpretation of changes in DNA methylation in relation to prognosis. These robust criteria allowed us to select a sample of patients with community-acquired infections, ensuring that the only difference between cases and controls was the presence or absence of sepsis.

The median patient age was 78, with 85% aged ≥65, reflecting the increased sepsis risk in older populations (Mayr et al., 2014). Women comprised 58.3% of the sample, which may be attributed to UTI being the most frequent source of infection (Artero et al., 2012). Comorbidity was high, with a median Charlson Comorbidity Index of 5. Frailty and dependency levels were notable: 30% were frail, 31.1% pre-frail, and 30% required some assistance with activities of daily living (ADL). These findings align with other sepsis studies (Kopczynska et al., 2018; Cox et al., 2020; Li et al., 2023), highlighting the vulnerability of this population. There were no major differences in baseline characteristics between the sepsis and non-sepsis groups, except for a higher prevalence of a history of neoplasia (20% vs. 3.3%) and malnutrition risk (43.3% vs. 16.7%) in the sepsis group. Mortality was low at 3.3%, in contrast to literature estimates ranging from 10% to 52% (Vardi et al., 2013; Rowe and McKoy, 2017), likely due to the study design, which excluded patients with baseline conditions leading to a short life expectancy to minimize bias.

Significant differences in DNA methylation patterns between septic and non-septic patients were identified. Specifically, 4,640 DMPs and 85 DMRs were found. Genes related to immune response, particularly neutrophil-mediated immunity and bactericidal granule components, showed prominent changes. Other relevant processes included monocyte, Th2 lymphocyte differentiation and immunoglobulin production regulation.

It is noteworthy that the sepsis group had a higher percentage of patients at risk of malnutrition based on their MNA scale scores. Although the MNA scale is a nutritional screening tool and does not define malnutrition on its own, it could be associated with nutritional deficiencies such as low levels of folic acid and vitamin B12. These deficiencies may impact the DNA methylation pattern by reducing the availability of S-adenosylmethionine, the main donor of methyl groups. We know that the resulting hypomethylation can alter gene expression and contribute to various pathologies, including neurodegenerative diseases, cardiovascular disorders, and cancer (García-Giménez et al., 2014; Kok et al., 2015; Monasso et al., 2021). Although our study does not analyze these aspects, a question that arises for future research is whether malnourished patients had lower baseline methylation levels, thereby promoting greater hypomethylation that could contribute to the development of sepsis.

We reviewed and identified those genes with a high differential methylation level methylation (DMPs with a β-value >8% and DMRs with a β-value >5%). Subsequently, we conducted a search for those genes that played a significant role in molecular or cellular pathways involved in the immune response, as identified in the enrichment analysis. The candidate genes were further investigated in terms of their biological relevance to sepsis, based on the various biological processes they were involved in and previous findings reported by other authors. Finally, the genes considered to have the greatest potential as biomarkers were selected.

Four genes (*AZU1*, *MPO*, *SERPINA1*, and *SLX4*) were selected for validation based on enrichment analyses. All four genes showed hypomethylation in septic patients, indicating increased protein transcription. *AZU1* encodes azurocidin, a critical antimicrobial protein linked to bacterial clearance and early immune activation. A recent meta-analysis that included 26 studies with 3668 patients found that the expression of azurocidin had a combined sensitivity of 0.85 (95% CI 0.79-0.90) and a specificity of 0.91 (95% CI 0.82-0.96), being superior to procalcitonin and C-reactive protein. In addition, a significant increase in plasma levels was observed at least 24h before the diagnosis of sepsis (Wu et al., 2021).

*MPO* plays a central role in neutrophil antimicrobial activity by producing hypochlorous acid to eliminate pathogens. Its expression levels have been shown to correlate with sepsis severity and inflammatory outcomes. In the study by Schrijver et al., higher levels of MPO were observed in patients with sepsis and septic shock compared with patients without sepsis and patients with noninfectious SIRS (60 ng/ml vs. 43 ng/ml, P = 0.002). Furthermore, MPO levels were associated with 30-day mortality (p = 0.032), and higher levels were associated with higher mortality and higher scores on the SOFA and APACHE-IV scales (Schrijver et al., 2017).

*SLX4* is involved in maintaining genomic integrity during oxidative stress and immune activation. Its modulation during sepsis highlights its relevance in cellular repair mechanisms and immune response. In a study to discover sepsis-specific plasma peptides, this protein was found to be significantly elevated in sepsis patients (Thavarajah et al., 2020).

*SERPINA1* encodes alpha-1 antitrypsin, a key anti-inflammatory protein that regulates neutrophil protease activity, reducing tissue damage during inflammatory processes. Its upregulation in septic patients reflects its role in immune regulation (Kalsheker et al., 2002). Some studies have analyzed SERPINA1 expression as a potential biomarker in sepsis. Zhou et al. found a close relationship with sepsis in pediatric patients (Zhou et al., 2021), but it was not included in the final selection as a diagnostic test. On the other hand, Yang et al., after preprocessing data from the mRNA expression profile in 70 samples from adult patients, selected several genes with a potential use as a biomarker among which SERPINA1 was found (Yang and Li, 2017).

At the clinical level, this study found that the degree of hypomethylation in the selected genes corresponds to the degree of inflammation and severity measured by clinical scoring systems. All genes showed a strong correlation with serum PCT levels and SOFA scores, as well as a moderate correlation with APACHE-II scores and albumin levels. The gene with the highest number of correlations was SERPINA1, which correlated with inflammatory markers such as CRP and PCT, severity scales like SOFA and APACHE-II, and moderately with vital signs such as mean arterial pressure and respiratory rate, as well as organ failure markers such as creatinine levels. These findings are consistent with classical literature, as it is well known that AAT (the protein encoded by the SERPINA1 gene) is upregulated during the acute phase of inflammatory processes (Lisowska-Myjak, 2005). Moreover, in a small study involving seven patients, serum levels of this protein was identified as a potentially useful biomarker for differentiating sepsis patients, particularly those with severe presentations accompanied by consumptive coagulopathy (He et al., 2014). Overall, these findings underscore the potential of DNA methylation as biomarkers in sepsis, particularly for *AZU1*, *MPO*, *SERPINA1*, and *SLX4*, which show strong correlations with clinical indicators of severity and inflammation. These results suggest that their well-established roles in immune mechanisms translate into clinically identifiable effects.

For these reasons, utility of these epigenetic marks for diagnosing sepsis and predicting poor prognosis was analyzed. Since they have not been previously described, their optimal cut-off points are unknown. Thus, we assessed the AUC for predicting poor prognosis for these genes alongside PCT and clinical scales (qSOFA, SOFA, and APACHE-II). PCT and clinical scales performed well, with APACHE-II showing the best result (AUC 0.796). Notably, *SLX4* and *MPO* showed slight improvements in predicting poor prognosis compared to the clinical scales (AUC 0.821 and 0.801, respectively).

We also compared the diagnostic capability of the methylation levels in *SERPINA1*, *AZU1*, *MPO*, and *SLX4* with commonly used inflammatory markers, particularly CRP and PCT. All epigenetic marks demonstrated good diagnostic capability for sepsis, surpassing CRP. However, in our study PCT was the best biomarker for sepsis diagnosis (AUC 0.925), followed by *SERPINA1* (AUC 0.858). The results for CRP and PCT align with previous studies (Mustafić et al., 2018; Tan et al., 2019), suggesting generalizability of our findings, though further validation is required.

It is noteworthy that sepsis is associated with global DNA hypomethylation. In previous work published by our group, we identified 1,256 differentially methylated regions (DMRs), of which 798 were hypomethylated and 458 were hypermethylated when comparing septic patients to critically ill patients without sepsis (Beltrán-García et al., 2024).

This study provides novel, unpublished insights into epigenetic changes in white blood cells obtained from septic patients, including the first epigenome-wide DNA methylation analysis from whole blood samples from septic and non-septic infected adults admitted in the internal medicine department of a tertiary hospital. The results obtained provides valuable information about the DNA methylation changes occurring as consequence of the development of a septic process but importantly, highlight the potential of DNA methylation as a diagnostic and prognostic biomarker. To our knowledge, only three clinical studies have analyzed DNA methylation in adult septic patients. As previously indicated, Binnie et al. (2020) identified 56 differentially methylated genes associated with sepsis, including *MPO*. Functional analysis showed increased methyltransferase activity, cell adhesion, and antigen presentation, correlating with clinical severity and hospital stay. Other study by Lorente-Sorolla et al., focused on monocytic DNA methylation changes in sepsis (n=18) and their association with a tolerized phenotype, correlating them with IL-10 and IL-6 levels and SOFA scores (Lorente-Sorolla et al., 2019). The most recent study, analyzed DNA methylation in leukocytes from patients (n=12) admitted in the ICU with sepsis and septic shock and compared the results with the DNA methylation profile of patients with intracranial hemorrhage (Beltrán-García et al., 2024). The most remarkable differences in methylation were found in immunosuppression related genes *IL10*, *S100A8*, and *TREM1*, the pro-inflammatory inflammasome-related interleukin *IL1B*, and *TNFAIP8* gene, which is also related with immunomodulatory functions. Interestingly, the methylation status of this genes was associated to organ dysfunction and lactate levels. However, none of them evaluated the diagnostic or prognostic usefulness of DNA methylation changes.

The scarce number of clinical studies analyzing epigenomic changes in sepsis usually compare patients with sepsis to those with other acute/critical conditions. Therefore, our findings are particularly insightful at a pathophysiological level. Additionally, from a clinical perspective, they may hold greater utility, as current diagnostic criteria for sepsis continue to rely on clinical scoring systems with suboptimal degrees of sensitivity and specificity. This highlights the need to find new, accurate biomarkers to identify patients whose infections have already caused immune dysregulation, which may lead to worse outcomes.

The study’s primary limitation is its small sample size, influenced by strict inclusion criteria and COVID-19 pandemic recruitment challenges. The single-center design and prevalence of UTI may affect the generalizability. Despite these constraints, rigorous patient selection and a second cohort for validation enhance the results’ reliability. Use of whole blood samples, while limiting sensitivity to minor cell subtype changes, remains a practical choice for biomarker studies. Most studies on sepsis make comparisons with uninfected patients. Comparing sepsis patients to those with the similar infections but without sepsis provides valuable insights into early sepsis identification and immune dysregulation. Our approach creates a highly homogeneous cohort, reducing many confounding factors. Overall, the findings are robust and have practical implications for clinical application.

To sum up, these findings reveal extensive DNA hypomethylation in sepsis patients compared to those with the same type of infections who do not develop sepsis. DNA hypomethylation mainly affect genes involved in immune regulation. *SERPINA1*, *AZU1*, *MPO* and *SLX4* genes stand out for their correlation with inflammation and severity and have potential as biomarkers for diagnosing sepsis and predicting poor prognosis.

Importantly, the determination of the methylation levels of these four genes using bisulfite pyrosequencing offers a practical and cost-effective approach for clinical application, with the potential to serve as robust diagnostic and prognostic biomarkers for sepsis, enabling earlier detection and personalized treatment strategies.

## Data Availability

The datasets presented in this study can be found in online repositories. The accession number in the GEO (Gene Expression Omnibus) repository is GSE285813.

## Supplementary material

Supplementary Figure 1 Flowchart of the study population.
