# Supplementary figures and images for: Epigenome-wide DNA methylation profiling in septic and non-septic patients with similar infections: potential use as sepsis biomarkers
Source: Front Cell Infect Microbiol. 2025 Jan 24;14:1532417. doi: 10.3389/fcimb.2024.1532417 (PMC11802815; doi:10.3389/fcimb.2024.1532417)

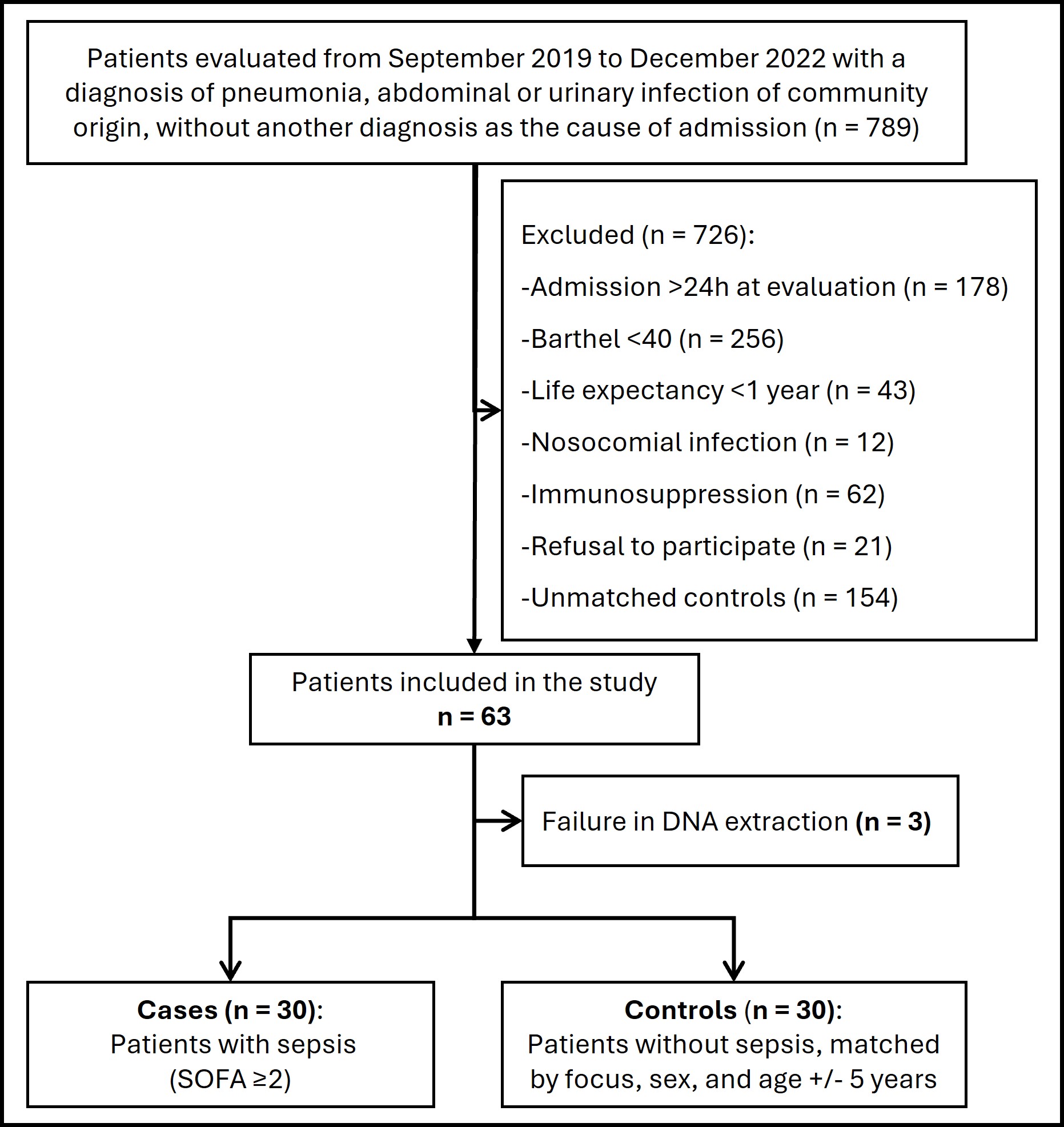

Supplement: Supplementary Figure 1 — Flowchart of the study population. [file DataSheet1.zip › Figure S1.jpg]
